# Removal of Ring, from a Finger

**Published:** 1853-11

**Authors:** A. C. Castle


### *Removal of a Ring from a Young Lady's Finger.*

BY A. C. CASTLE, M. D.

An interesting young lady, about 17 years of age, had presented to her a gold ring, which she forced over the joints of her middle finger. After a few minutes the finger commenced swelling, and the ring could not be removed. The family physitian, Dr. ——, was sent for, but could do nothing. The family, and the young lady especially, were now in the greatest consternation. A jeweler was sent for. After many futile attempts to cut the ring with cutting nippers, and to saw it apart with a fine saw, and after bruising and lascerating the flesh, warm fomentations and leeches were applied, but all without affording the slightest benefit. Dr. —— requested my presence, with the compliment that "perhaps my mechanical ingenuity might suggest something." I at once proceeded to the house of the patient, and found the young lady in the most deplorable state of mental agony, the doctor embarrassed, and the family in a high state of excitement. I procured some prepared chalk, and applied it between the ridges of swollen flesh, and all round the finger, and succeeded in drying the oozing and abraded flesh; then with a narrow piece of soft linen I succeeded in polishing the ring, by drawing it gently round the finger between the swollen parts. I then apylied *quicksilver* to the whole surface of the ring. *In less than three minutes* the ring was *broken* (by pressing it together) in four pieces, to the great relief of all parties.

In a similar manner, without the chalk, I sometime since extracted a small brass ring from the ear of a child, who, child-like, had inserted it into the cavity of the ear. The operation was more painful and tedious, but was equally successful.

THE MODUS OPERANDI.—The quicksilver at once permeates the metals, *if clean*—with the exception of iron, steel, platina, and one or two others—and amalgamates with them. It immediately crystallizes and renders the metal as hard and as brittle as glass. Hence the ease with which metals amalgamated with quicksilver can be broken. —*Boston Med. and Surg. Jour.*

